# The Role of Personal and Political Values in Predicting Environmental Attitudes and Pro-environmental Behavior in Kazakhstan

**DOI:** 10.3389/fpsyg.2020.584292

**Published:** 2020-12-23

**Authors:** Fatikha Agissova, Elena Sautkina

**Affiliations:** School of Psychology, National Research University Higher School of Economics (HSE University), Moscow, Russia

**Keywords:** personal values, political values, environmental concern, new environmental paradigm, pro-environmental behavior, post-Soviet states, Kazakhstan, developing world

## Abstract

Although it is widely accepted that personal values of Self-Transcendence are a positive predictor of environmentalism, and Self-Enhancement values are a negative one, these results are not conclusive for all cultural contexts. Regarding political ideologies, research concludes that liberals tend to be more concerned about the environment than conservatives. However, this two-dimensional take on political ideologies does not grasp the diversity of political views, which could be achieved by focusing on political values. In this research, we studied the role of personal and political values in predicting environmental attitudes and behavior in Kazakhstan, a developing country in Central Asia. Using an online survey (*n* = 305), we found that Security was a strong predictor of both environmental concern and New Environmental Paradigm (NEP), overshadowing the effect of traditionally accepted value dimensions of Self-Transcendence and Self-Enhancement. While Self-Direction positively predicted environmental concern, Universalism and Benevolence were positive predictors of NEP. Among political values, Civil Liberties predicted NEP positively, and had no significant effect on environmental concern, while Free Enterprise predicted environmental concern negatively, and had no significant effect on NEP. Environmental concern was a strong predictor of all pro-environmental behaviors included in the study (littering, recycling, environmental citizenship, and community action), fully mediating the effect of NEP. Based on personal and political values, three profiles of Kazakhstanis who engaged differently in pro-environmental behavior were identified.

## Introduction

There is a wide range of pressing environmental issues in Kazakhstan, a post-Soviet country located in Central Asia, with a population of 18.78 million and a GDP per capita of US $9,731 (The World Bank Open Data, 2019). The ethnic majority are Kazakhs (over 63%); other ethnicities include Russians (23%), Uzbeks (2.9%), Ukrainians (2.1%), Uighurs (1.4%), Tatars (1.3%), and Germans (1.1%). The country’s main religion, Islam, is practiced by over 70% of the population (World Population Review, 2020). The environmental issues in this country include a deterioration of land resources, as well as air, soil, and water pollution, and an increasing water scarcity, including due to oil, gas, and metal extraction activities (Onyusheva et al., 2018; Russell et al., 2018). These problems pose a large threat to numerous aspects of the people’s lives in Kazakhstan. For example, it is estimated that water scarcity will reach 70% by 2050. Water levels show threatening tendencies in the Caspian Sea and Balkhash, while the shrinking of the Aral Sea, also located in Kazakhstan, is known internationally as one of the largest human-made ecological disasters (Micklin, 2010; Onyusheva et al., 2018). These issues can be partly attributed to poor environmental performance of Kazakhstan which, according to the Environmental Performance Index, occupies the 85th position among 180 countries (Wendling et al., 2020). Therefore, it is necessary to raise people’s awareness about the environmental issues and promote pro-environmental behavior, as well as improve the state of environmental policy in this country.

Environmental psychology research has extensively studied values and political ideologies as important predictors of environmentalism (Stern and Dietz, 1994; Cruz, 2017). Personal values are important socio-cultural predictors, as they show what is considered desirable in a particular culture (Schwartz, 1992). While there is a substantial body of research on the role of values, their role, especially in non-Western countries, have not been studied enough. Specifically, studies emphasize the role of Self-Transcendence Vs. Self-Enhancement dimension in predicting pro-environmental attitudes and behaviors. However, a lack of cross-cultural evidence in this area was shown in a meta-analysis by Hurst et al. (2013). While the relationship between materialistic values, including Schwartz’s Self-Enhancement dimension, and environmentalism was significant among the Western nations, in Chile, the only non-Western country included in their analysis, these values did not have a significant association with environmental attitudes. Another study, conducted in Egypt, showed that the values of Tradition and Islamic religiosity predicted environmentalism positively (Rice, 2006).

Political values, which reflect people’s ideas about the desired political course of the country (Schwartz et al., 2010), have emerged as two distinct political ideologies, liberalism and conservatism, divided on the issue of environmentalism and climate change (McCright and Dunlap, 2011; Clayton et al., 2015; Hornsey et al., 2016). This political polarization does not exist in Kazakhstan, a country with a single ruling party and a so-called “soft-authoritarian” political regime (Schatz and Maltseva, 2012). In this connection, Kazakhstan represents an interesting case for studying the relationship between political ideologies and environmental concern in a polarization-free context. Considering both the absence of political polarization in Kazakhstan and the lack of knowledge among Kazakhstanis about Western political ideologies, we decided to investigate the role of core political values as predictors of environmental attitudes and pro-environmental behavior.

We combined the study of Personal and Political Values to analyze and compare the role of the two important aspects of a nation’s value system in determining people’s environmental attitudes and behaviors. As studies of the relationship between environmental attitudes and pro-environmental behavior have produced mixed results (Tam and Chan, 2017), we decided to test whether this relationship was significant in Kazakhstan. In the current research, we chose four types of pro-environmental behavior that we consider specifically related to environmentalism in this country: littering, recycling, environmental citizenship, and environmental community action. As shown recently in two studies conducted in Russia (another post-Soviet country, similar in its context), several behavior types traditionally considered as pro-environmental in the West (e.g., frugal and transport behaviors) are unrelated or weakly related to environmental concern and are not seen as pro-environmental as such (Sautkina, 2019; Ivanova et al., 2020). Therefore, we have chosen to include in the present research behavior types that vary in difficulty (Kaiser et al., 2010) and are normally unlikely to be performed for other reasons than the environmental ones.

### Environmental Attitudes and Pro-environmental Behavior

Environmental concern is the attitude toward the seriousness of environmental problems (Dunlap and Jones, 2002). The meta-analysis by Hornsey et al. (2016) revealed that environmental concern measured by New Environmental Paradigm (NEP) is the strongest predictor of belief in climate change. However, NEP and belief in climate change do not necessarily lead to pro-environmental behavior (Hornsey et al., 2016). As stated by Berenguer et al. (2005), specific environmental concern, not NEP, can be more predictive of pro-environmental behavior (Berenguer et al., 2005). A limited amount of studies of public environmental concern in Kazakhstan showed very high rates of people’s concern, for example, already in early 2000s, Kazakhstanis perceived a significant decline in environmental conditions (Soltys and Orynbassarova, 2013). In this connection, we consider that for the context of Kazakhstan, the effect of environmental concern, rather than NEP, on pro-environmental behavior will be more significant. Nevertheless, a high degree of environmental concern does not necessarily translate in action to protect the environment. This environmental attitude–behavior gap was noted in studies of psychological barriers which hinder pro-environmental behavior (Lorenzoni et al., 2007; Gifford, 2011).

Pro-environmental behavior can be defined as an action that is consciously intended to benefit or reduce harm to the environment (Steg and Vlek, 2009). In this study, four types of pro-environmental behavior will be studied: Littering (consciously avoiding littering in outdoor public places), Recycling (sorting household waste such as plastic, glass, paper, etc.), Environmental Citizenship (i.e., communicating with others on environmental issues, learning about the environmental issues), and Environmental Community Action (voluntary participation in community clean-up and greening events).

### Values as Predictors of Environmental Concern and Pro-environmental Behavior

Schwartz defined values as belief systems that help people guide and evaluate their and others’ behavior. Ten values with distinct motivations (Schwartz, 1994) were divided into two motivational dimensions—Openness to Change vs. Conservation, and Self-Enhancement vs. Self-Transcendence. The universal nature of values and their equivalence across all the studied cultures have been proven (Schwartz, 1992).

Being a universal dimension, values have been found to predict pro-environmental behaviors (Poortinga et al., 2004; Lind et al., 2015). There is a large body of research, including meta-analytical evidence, suggesting that the Self-Enhancement values are related negatively, while Self-Transcendence values are related positively to environmental attitudes and pro-environmental behaviors (Stern and Dietz, 1994; Poortinga et al., 2004; Hurst et al., 2013; Klöckner, 2013). However, these findings were not replicated in other contexts, for example, more recent studies conducted in Russia found that Altruistic values were not related to environmentalism (Sautkina, 2019; Ünal et al., 2019).

Openness to Change vs. Conservation dimension held a weaker predictive power, but the studies indicated a negative effect of the value of Tradition on environmental attitudes, as well as on belief in adverse consequences of environmental degradation for self and the biosphere, therefore, indirectly negatively influencing pro-environmental behavior (Stern and Dietz, 1994; Schultz and Zelezny, 1999). These relationships were not found in Asian collectivist cultures. For example, an international study found that traditional values in Japan predicted environmentalism positively, while in the Netherlands and the United States, they predicted environmentalism negatively (Karp, 1996). In addition, the studied populations did not include Muslim cultures, as Islamic ideology emphasizes environmentalism (Yildirim, 2016). In this respect, Rice (2006) reported a strong correlation between the value of Tradition and religiosity in Egypt, as well as a significant positive effect of Tradition on public pro-environmental behavior. There is limited literature on the effect of another value from the Conservation dimension—Security. A poll conducted in Kazakhstan in 2007 showed that 71% of Kazakhstani respondents believed that environmental problems were damaging the health of their families (Freedman, 2009). Considering Kazakhstan’s position as a Central Asian country with a Muslim majority, and with alarmingly declining state of the environment, we can expect that the dimension of Conservation would be positively related to environmental attitudes and pro-environmental behaviors.

It is important to mention that most research work in this area has been carried out nearly 20 years ago. In addition, it has mainly been focusing on the North-American (mainly the US) context, little being known about other cultural, geographical, and political contexts. Meanwhile, the new evidence regarding other contexts is emerging. For instance, a recent study that surveyed Russian youth found a relationship between the value of Self-Direction, pro-environmental behavior, and environmental activism (Zibenberg et al., 2018). This finding reflects the recent generational changes in the perception of environmental issues, as well as the fact that environmentalism is becoming an important point of consideration for progressive youth in Russia. A close value type, Openness to Experience, was recently found to be positively associated with pro-environmental behavior, which was due to the fact that Openness is related to liberalism (Klein et al., 2019). In this connection, we assume that as environmental problems start receiving more attention and the consequences of climate and environmental change begin to affect people’s lives, the value basis that shapes environmental attitudes will be experiencing change, which we may have started to witness.

In the present study, we assume that the values that promote caring for other living beings and the world, i.e., the value dimension of Self-Transcendence, will be a positive predictor of environmental attitudes, while the egoistic value of Power, as in previous research, will remain a negative predictor. Openness to Change would be a positive predictor of environmental attitudes and behavior, consistent with Zibenberg et al. (2018) finding.

### Political Values as Predictors of Environmental Concern and Pro-environmental Behavior

Political polarization on environmental issues has been observed persistently over the past decades in the United States (McCright and Dunlap, 2011). A meta-analysis by Cruz (2017) found that political ideology significantly predicts environmental concern. In addition, liberal ideology directly positively predicted pro-environmental behaviors (Cruz, 2017). However, a closer analysis of different types of pro-environmental behavior showed that liberalism significantly predicts only public-sphere, environmental activism and citizenship behaviors, while having a weak association with private-sphere and environmental protection behaviors (Newman and Fernandes, 2016). However, these studies were mostly conducted in the United States, and the evidence of the relationship between environmental concern and political ideology in other cultural contexts is mixed: this association was not found to be consistent across different nations (Nawrotzki, 2012; Kim and Shin, 2015). These findings were supported by another research conducted in Germany and China, where people, regardless of political ideology, were concerned about the environment (Ziegler, 2017). Thus, the evidence suggests that the link between political ideology and environmental concern depends on the cultural context.

The political space of Kazakhstan drastically differs from those of the Western democracies. As evidenced by Bowyer (2008), it is virtually a one-party system where the conditions for the development of other parties and political competition are absent. In this system, the President’s party—Nur Otan—has the majority of seats in the Parliament and has no rivals (Bowyer, 2008). In such conditions, it is difficult to form, and identify with, a certain political ideology. Therefore, we assume that political views of Kazakhstanis do not conform to certain ideologies, but rather exist as separate value-based opinion clashes. Therefore, in this research, we chose a different construct that reflects political ideologies—the core political values.

The core political values are overarching ideas and beliefs about the proper functioning of the government, citizenship, and society, which influences choices, such as voting behavior (Schwartz et al., 2010). Schwartz et al. (2010) identified eight core political values: Law and Order, Traditional Morality, Equality, Free Enterprise, Civil Liberties, Blind Patriotism, Accepting Immigrants, and Foreign Military Intervention. These core political values correspond to basic personal values. As such, Traditional Morality, Blind Patriotism, and Law and Order represent people who strive for certainty, predictability, and preservation of social order, and reflect Schwartz’s value dimension of Conservation (Tradition and Security). In contrast to them, Accepting Immigrants reflects people’s readiness to endorse and accept what is different, which corresponds to the values of Openness to Change (Self-Direction and Stimulation). Free Enterprise, on the other hand, is related to Self-Enhancement (Power, Achievement, and Hedonism), emphasizing personal gain and achievement. Finally, Equality and Civil Liberties, representing care about the welfare of others, corresponds to the dimension of Self-Transcendence (Universalism and Benevolence).

To our knowledge, the relationships between the core political values and pro-environmental attitudes and behavior has not been looked at in the past, and the present research aims at filling this evidence gap.

### Defining Clusters Based on Values and Pro-environmental Behavior

Cluster analysis has been used in the past to identify groups of people that differ according to their values or behaviors, value types determining people’s behavior. For example, Lee et al. (2011) found four clusters of people with Self-Transcendence, Self-Enhancement, Openness to Change, and Conservation values in the United States and China, who demonstrated different travel choices.

Clusters can also be distinguished based on pro-environmental behaviors. In a study by Lind et al. (2015), three groups of people with different travel mode choices were identified: those who use public transport, private car, and who prefer to walk. In another study, clusters were identified based on preferences for tourism. The three clusters included typical, economical, and sustainable tourists, who represented different socio-demographic groups and had different scores on environmental values (Holmes et al., 2019). Also, a cluster analysis comparing groups of people differing in recycling behavior found non-recyclers, convinced recyclers, and potential recyclers, who had different levels of environmental concern and awareness of negative consequences of not recycling (Elgaaied, 2012).

In this research, we consider how clusters based on personal and core political values differ in terms of their pro-environmental behavior.

### Research Hypotheses

Based on our theoretical review, we have the following hypotheses for this study:

H1. Environmental Attitudes (Environmental Concern and NEP) will positively predict Pro-Environmental Behavior.

H2. Personal values of Self-Transcendence (Universalism, Benevolence); Openness to Change (Stimulation, Self-Direction) and Achievement; Conservation (Tradition, Security); Core political values of Equality, Civil Liberties, and Immigration; and Blind Patriotism, Law and Order, and Traditional Morality will positively predict Environmental Attitudes.

H3. Personal values of Self-Enhancement (Power, Hedonism) and core political value of Free Enterprise will negatively predict Environmental Attitudes.

H4. Personal values of Self-Transcendence (Universalism, Benevolence); Openness to Change (Stimulation, Self-Direction) and Achievement; Conservation (Tradition, Security); Core political values of Equality, Civil Liberties, Immigration, Blind Patriotism, Law and Order, and Traditional Morality will positively predict Pro-Environmental Behavior.

H5. Personal values of Self-Enhancement (Power, Hedonism) and core political value of Free Enterprise will negatively predict Pro-Environmental Behavior.

**FIGURE 1:**
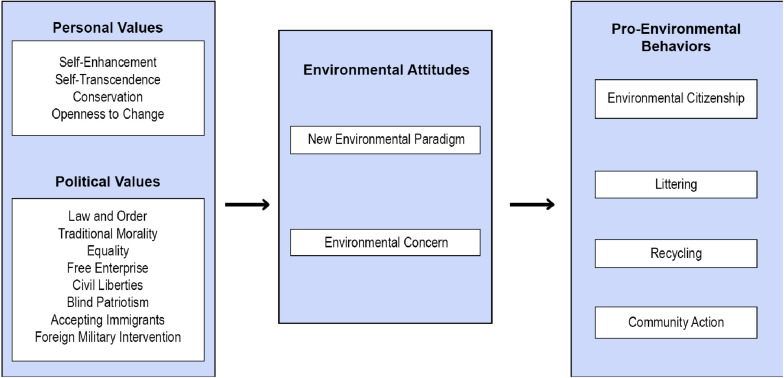
Theoretical model: relationships between values, environmental attitudes, and pro-environmental behaviors.

Figure 1 presents the theoretical model based on the hypotheses.

## Materials and Methods

### Data Collection

#### Sampling

Data were collected using the method of snowball sampling by distributing the questionnaires through social media platforms from December 25, 2019 to February 7, 2020. The questionnaires were created in Google forms in two languages: Kazakh and Russian, the two official languages in Kazakhstan. To avoid the self-selection bias related to environmentalism, participants were asked to fill in a survey about “political psychology of Kazakhstanis.” In the first page of the form, the participants were informed that they could quit the survey at any moment and that their personal information would not be collected. The questionnaires were presented in Kazakh and Russian languages. The research design was approved by the Institutional Review Board of the National Research University Higher School of Economics, Russia.

The sample size was established based on power analysis with medium effect size *f*^2^ = 0.15, *a* = 0.05, and power = 0.95. With a number of predictors set at 20, the minimum sample size required was *N* = 222. A smaller effect size was only found for regression of recycling behavior; however, a *post-hoc* power analysis showed that with *N* = 305 and power = 0.80, an effect size of *f*^2^ = 0.09 can be achieved.

The final sample included 305 respondents, 114 of them responded to the Kazakh version of the questionnaire (37.4%). The sample was well-balanced on gender (60% females) and was relatively young with 54% of the respondents aged 18–24, 33% aged 25–44, and 13% over 45. Participants were mostly educated, the vast majority having higher education (64%) or incomplete higher education (19%), with some respondents having secondary (9%) and secondary vocational (6%) education level. Most of the respondents were ethnic Kazakhs (87%) followed by Russians (7%), while other ethnicities (e.g., Tatars, Ukrainians, Uighurs, ethnic Koreans, and ethnic Germans) accounted for less than 1% per category. Respondents lived in the main big cities of Astana, Almaty, and Shymkent (40%), followed by residents of regional centers (25%) and small and medium towns (22%), with fewer living in villages (13%). Most of the answers came from the Northern part of the country—Akmola (24%) and Kostanay (6%) regions, its Western part—West Kazakhstan (33%) and Atyrau (9%) regions, as well as Almaty region (12%), located in the South-East of Kazakhstan.

### Research Instrument

The survey included the scales of basic personal values, core political values, new environmental paradigm (NEP), environmental concern (EC), pro-environmental behaviors (littering, recycling, environmental citizenship, community action), and socio-demographic variables (age, gender, education, region, settlement size, nationality).

#### Scale Translation

A rigorous back-translation process was used to prepare the Kazakh version of all scales. The questionnaires were translated independently by professional translators, native Kazakh speakers, into Kazakh and back into English. Original and translated versions of the scale were compared by a native English speaker with a degree in Psychology. Problematic items were discussed and changed following a discussion with a third professional English translator, a native Kazakh speaker. Further, the scales were administered to 45 Kazakhs, and one cognitive interview was conducted. Despite the difficulty of understanding of some questions due to the peculiarities of the language, the scales showed good reliability. The Kazakh and Russian versions of the questionnaires used in the study are presented in the Supplementary File 3.

##### Independent variables

###### Personal values

The basic personal values were measured using Short Schwartz’s Value Survey containing 10 items reflecting each of 10 Schwartz’s values. The survey was proven to have external and internal validity and reliability (Lindeman and Verkasalo, 2005). The item of Universalism (broadmindedness, beauty of nature and arts, social justice, a world at peace, equality, wisdom, unity with nature, environmental protection) was modified to avoid correlation with dependent variables due to environment-related descriptions in the parentheses. We removed the descriptions “beauty of nature and arts,” “unity with nature,” and “environmental protection.” This left five descriptions of the value. Respondents rated each value on a scale ranging from 0 (opposed to my principles), 1 (not important), 4 (important) to 8 (of supreme importance).

###### Political values

The scale of Core Political Values (Schwartz et al., 2010), which has an adapted Russian version (Kholod, 2016) and contains 34 questions, was used. The scale measures the core political values of Equality, Free Enterprise, Traditional Morality, Law and Order, Blind Patriotism, Civil Liberties, attitudes toward Accepting Immigrants, and Foreign Military Intervention. Responses range from 1 (completely disagree) to 5 (completely agree).

##### Dependent variables

*Environmental concern (EC)* was measured using a three-item scale (Sautkina, 2019) adapted from Berenguer et al. (2005) and included the following questions: *“To what extent are you concerned about the environment in your region?,” “To what extent are you concerned about the environment in your country?,”* and *“To what extent are you concerned about the environment in the world?”* The answers ranged on a 7-point scale from 1 (not at all) to 7 (totally). The scale has previously shown a good internal validity and reliability (Sautkina, 2019; Ivanova et al., 2020).

*New Environmental Paradigm (NEP)* scale consists of 15 statements for agreement or disagreement (Dunlap et al., 2000). The answers range on a 5-point scale from 1 (completely disagree) to 5 (completely agree). The adapted Russian version of the scale was used (Kryazh, 2013).

###### Pro-environmental behavior

Four types of pro-environmental behavior (littering, recycling, environmental citizenship, community action) were measured using self-report questions, adapted from different existing scales of pro-environmental behavior. The behavior types were chosen considering, in Kazakhstan, the availability of facilities to perform them and the awareness about their environmental character.

For Littering and Recycling, participants were asked to mark how often they performed the following activities: *“Carrying litter with oneself until one finds a bin”* (Zero Waste Scotland, 2015), *“In car or boat, throwing things out on the highways or waterways”* (reversed item from Schultz, 2009), *“Recycling dead batteries,”* and *“Taking paper/newspapers/magazines, glass bottles/jars/glass, plastic bottles/plastic packaging to recycling”* (Kaiser, 1998).

For Environmental Citizenship, participants were asked to mark how often they performed the following activities in the last 6 months (Alisat and Riemer, 2015): *“Talked with others about environmental issues [e.g., spouse, partner, parent(s), children, or friends]”* and *“Used online tools (e.g., YouTube, Facebook, Wikipedia) to raise awareness about environmental issues.”* A single item from Alisat and Riemer (2015) was used to measure Community Action: *“Participated in nature conservation efforts (e.g., planting trees, restoration of waterways).”* Responses for all questions ranged from 1 (never) to 5 (always).

### Analytical Procedures

Exploratory (EFA) and confirmatory factor analyses (CFA) were used to determine if the structure of the translated political values scale corresponded to the original structure by Schwartz et al. (2010). We assessed the overall model fit using chi-square divided by the degrees of freedom (χ^2^/df), the Comparative Fit Index (CFI, with an acceptable CFI ≥ 0.9), standardized root mean square residual (SRMR, with an acceptable SRMR < 0.08), and the root mean square error of approximation (RMSEA, with an acceptable RMSEA < 0.08; Hu and Bentler, 1999). SPSS AMOS 22.0.0 was used for fitting the models. For estimating the internal reliability of scales containing more than one item, Cronbach’s alpha was used. For initial assessment of the data, the Kolmogorov–Smirnov tests of normality, descriptive statistics, and correlation analysis were performed. To compare socio-demographic groups, Mann–Whitney U for two non-normal independent samples was used for gender and language groups, and ANOVA was used for settlement size and age groups. A multiple regression analysis was used to test the relationships between the variables. Structural Equation Modeling using SPSS AMOS 22.0.0 was used for fitting the hypothesized model in accordance with the acceptable criteria (Hu and Bentler, 1999). K-means cluster analysis was used to create value-based clusters. The differences between the groups were estimated using the analysis of variance test (ANOVA) and Bonferroni’s test. SPSS 22.0.0 Statistical Package was used to perform the analyses.

## Results

This section presents the analysis of a total of 305 questionnaires completed in Russian and Kazakh language in the following order: establishing reliability and equivalence, descriptive statistics and mean differences between socio-demographic groups, regression analysis testing the prediction hypotheses, and cluster analysis.

### Establishing Measure Reliability and Equivalence

#### Core Political Values

To determine if the structure of political values corresponded to the original structure (Schwartz et al., 2010), we performed an exploratory factor analysis with oblique Promax rotation using principal axis factoring, as in the original study. We have set the fixed number of factors to eight. According to the Kaiser–Meyer–Olkin, KMO = 0.86, the sampling was adequate to perform an EFA (Field, 2009). The factors explained 44.86% of variance. The EFA revealed that Traditional Morality and Blind Patriotism fall within a single factor and several items within factors had very low factor loadings. It was decided to delete items with factor loadings below 0.3. The items include Free Enterprise *“There should be more incentives for individual initiative even if this reduces equality in the distribution of wealth,”* Traditional Morality *“The right to life has to be guaranteed by law from the moment of conception,”* Foreign Military Intervention *“War is never justified,” “Going to war is sometimes the only solution to international problems,”* and *“Any act is justified to fight terrorism.”*

Two structures were tested using CFA with maximum likelihood estimation: with Traditional Morality and Blind Patriotism as separate factors (8-factor model) and as a single factor (7-factor model). The 7-factor model did not meet the CFA and SRMR criteria for acceptable fit to the data (χ^2^/df = 2.189; CFI = 0.848; RMSEA = 0.063; SRMR = 0.081; *p* < 0.001). The 8-factor model showed a better fit to the data for all criteria (χ^2^/df = 2.097; CFI = 0.863; RMSEA = 0.060; SRMR = 0.079; *p* < 0.001). Though CFI did not meet the threshold criteria (CFI > 0.9), it was decided not to correlate residual errors following the modification indices, as this is theoretically unjustifiable (Hermida, 2015). However, Traditional Morality and Blind Patriotism showed low discriminant validity, correlating at 0.86, *p* < 0.001.

#### Internal Consistencies of the Scales

Cronbach’s alphas for the scales are presented in Supplementary Table 1. All scales except for Accepting Immigrants and Foreign Military Intervention, Environmental Citizenship, and Littering had acceptable internal consistency above 0.6, for the majority—above 0.7 and 0.8. For Accepting Immigrants, despite the low factor loading of the item *“People who come to live here from other countries generally take jobs away from Kazakhstani workers”* and an increase in Cronbach’s alpha to 0.58 when it is deleted, it was decided to leave the item as it made a meaningful contribution to understanding the core of immigration fear. Correlations between three items for Accepting Immigrants: *r* = 0.41, *p* < 0.001; with the reverse item: *r* = −0.16, *p* = 0.004; *r* = −0.09, *p* = 11, ns. Foreign Military Intervention (correlation between items: *r* = 0.32, *p* < 0.001), Environmental Citizenship (*r* = 0.38, *p* < 0.001), and Littering (*r* = −0.32, *p* < 0.001) had low internal consistencies because they included only two items. In addition, Littering scale had a reverse item. Considering the generally fairly acceptable levels of internal consistency, mean scores of variables were used in further analyses.

#### Equivalence of Internal Consistencies of Kazakh and Russian Language Versions

Internal consistencies of language subsamples were relatively equal for the scales of EC (α = 0.89 for Kazakh, α = 0.88 for Russian), Littering (α = 0.45 for Kazakh, α = 0.47 for Russian), Environmental Citizenship (α = 0.5 for Kazakh, α = 0.57 for Russian), and Recycling (α = 0.73 for Kazakh, α = 0.68 for Russian). NEP scale’s consistency was lower for the Kazakh language subsample (α = 0.42) than for the Russian subsample (α = 0.73). Following the analysis of means for each item and item correlations with EC, it was decided to delete items 4, 6, 8, 10, and 14: *“Human ingenuity will insure that we do not make the earth unlivable,” “The earth has plenty of natural resources if we just learn how to develop them,” “The balance of nature is strong enough to cope with the impacts of modern industrial nations,” “The so-called ‘ecological crisis’ facing humankind has been greatly exaggerated,”* and *“Humans will eventually learn enough about how nature works to be able to control it.”* Items 4, 6, 8, and 10 had significant positive correlation with EC, and item 14 did not significantly correlate with EC in the Kazakh language subsample. Deleting these items increased the internal consistency of the Kazakh version of the scale; Cronbach’s alpha of the 10-item version was equal and acceptable for both languages (see Supplementary File 1).

### Main Results

Means, SDs, and correlations between variables are presented in Supplementary Table 1. Socio-demographic differences between variables (Mann–Whitney U for gender and language, F for settlement size and age differences) are presented in Supplementary Table 2.

#### Results of Multiple Regression Analysis

In the regression analysis, considering the large number of predictors, variance inflation factor (VIF) and tolerance were calculated. Both VIF and tolerance did not exceed threshold levels above 10 and below 0.2, respectively (Field, 2009), which allows for further analysis.

Results of regression analysis of EC and NEP are presented in Table 1. Free Enterprise, Security, and Self-Direction were significant predictors of EC: people with higher values of Security and Self-Direction tended to have higher EC. Higher scores on Free Enterprise, on the other hand, decreased respondents’ EC. These findings partially confirm the hypotheses 2 and 3, which stated that personal values of Conservation and Openness to Change, and political values of Free Enterprise will have an effect on environmental attitudes.

**TABLE 1:** Regression of EC and NEP on political and personal values.

	EC^a^			NEP^b^		
		
	β	t	Semi-partial correlation	β	t	Semi-partial correlation
(Constant)		4.88***			7.55***	
Foreign military intervention	0.06	0.93	0.05	0.11	1.85	0.09
Free enterprise	–0.13	−2.23*	–0.11	–0.09	–1.66	–0.08
Traditional morality	0.08	1.05	0.05	0.15	1.92	0.09
Equality	0.10	1.54	0.08	0.09	1.54	0.08
Accepting immigrants	0.01	0.11	0.01	–0.01	–0.08	–0.00
Blind patriotism	–0.01	–0.15	–0.01	–0.13	–1.75	–0.09
Civil liberties	–0.00	–0.03	–0.00	0.16	2.45**	0.12
Law and order	–0.02	–0.35	–0.02	–0.10	–1.59	–0.08
Power	0.03	0.53	0.03	0.05	0.94	0.05
Achievement	0.01	0.09	0.00	0.01	0.13	0.01
Hedonism	–0.04	–0.62	–0.03	0.06	1.05	0.05
Stimulation	–0.05	–0.72	–0.04	0.01	0.07	0.00
Self-direction	0.18	2.34*	0.12	–0.07	–0.91	–0.05
Universalism	0.08	1.05	0.05	0.14	1.94*	0.10
Benevolence	0.07	0.89	0.05	0.20	2.64**	0.13
Tradition	–0.01	–0.14	–0.01	–0.11	–1.29	–0.06
Conformity	0.06	0.64	0.03	0.02	0.27	0.01
Security	0.20	2.67**	0.14	0.22	3.00**	0.15

R^2^	0.25			0.29		
Cohen’s f^2^	0.33			0.41		

*^*a*^EC, Environmental Concern.*

^*b*^NEP, New Environmental Paradigm.

***p < 0.001, **p < 0.01, *p < 0.05.

NEP was mainly predicted by Civil Liberties, Universalism, Benevolence, and Security—all of them tended to increase the levels of NEP. Therefore, these findings partially confirm hypothesis 2. The absence of effect of Self-Enhancement or of Free Enterprise values on NEP partially rejects hypothesis 3 regarding the NEP. Interestingly, while Free Enterprise was related negatively to EC, it did not have a significant relationship with NEP.

Table 2 presents results of regression analysis of pro-environmental behaviors taking into account the socio-demographic variables. All pro-environmental behaviors were significantly predicted by EC, but NEP did not predict any pro-environmental behavior significantly. This finding partially confirms hypothesis 1 with regard to the relationship between EC and pro-environmental behavior. Environmental Citizenship behavior was significantly predicted by Universalism (positively) and Conformity (negatively), meaning that people who held Universalist values tended to educate themselves and talk about the environment more, and people who valued Conformity tended to engage in this behavior less. This result was unexpected considering hypothesis 4 which stated that both Universalism and Conformity will be positive predictors of pro-environmental behavior. Confirming the prediction regarding the positive effect of Universalism, it shows an opposite negative association with Conformity. Reasons for such findings are discussed below. Regarding socio-demographic variables, age was negatively related to environmental citizenship: younger respondents tended to engage in this behavior more than the older respondents.

**TABLE 2:** Regression of pro-environmental behaviors on EC, NEP, and political and personal values.

	Environmental citizenship		Community action		Recycling		Littering	
				
	β	t	β	t	β	t	β	t
(Constant)		4.99***		3.10**		1.69		6.98***
Age	–0.14	−2.64**	0.21	4.04***	0.14	2.39**	–0.01	–0.01
Settlement size	–0.07	–1.21	0.07	1.33	0.01	0.13	0.01	0.20
Gender	–0.10	–1.79	–0.06	–1.10	–0.01	–0.22	0.15	2.89**
Language	–0.07	–1.23	–0.30	−5.69***	–0.07	–1.19	0.17	3.23***
EC^*a*^	0.45	7.22***	0.29	4.99***	0.16	2.47**	0.35	5.95***
NEP^*b*^	–0.03	–0.42	–0.06	–0.98	–0.07	–0.98	–0.03	–0.47
Civil liberties	−	−	−	−	−	−	0.12	2.15*
Accepting immigrants	−	−	−	−	0.11	1.95*	−	−
Power	−	−	0.12	2.33*	0.17	3.06**	−	−
Universalism	0.15	2.50**	−	−	−	−	−	−
Benevolence	−	−	−	−	−	−	0.13	2.37**
Conformity	–0.14	−2.27*	−	−	−	−	−	−
R^2^	0.23		0.26		0.08		0.27	
Cohen’s f^2^	0.30		0.34		0.09		0.38	

*^*a*^EC, Environmental Concern.*

^*b*^NEP, New Environmental Paradigm.

***p < 0.001; **p < 0.01; *p < 0.05.

Community Action behavior was predicted by Power, in addition to EC. People who scored higher on Power tended to participate more often in community action, such as community greening and clean-ups more than other respondents. These findings reject hypothesis 5, showing an opposite tendency. The value of Power did not decrease, as was expected, but increased Community Action behavior. Age and Language significantly predicted Community Action behavior, meaning that the older respondents and Kazakh-speaking respondents tended to engage in community action more than other socio-demographic groups.

The model predicted a very small amount of variance in Recycling. Recycling behavior was predicted by Power: people who valued Power tended to recycle more. This finding is in line with the result found for Community Action behavior: hypothesis 5 was rejected, giving a result opposite to the one expected. Instead of decreasing, Power increased recycling behavior. Among socio-demographic variables, age was positively related to Recycling. Older people tended to recycle more than other age groups.

Littering behavior was predicted by Civil Liberties and Benevolence positively, meaning that people who valued Civil Liberties and Benevolence tended to litter less. These findings partially confirm hypothesis 4, which stated that there will be positive relationships between these variables. Gender and Language positively predicted Littering, meaning that female and Russian-speaking respondents tended to litter less.

#### Testing the Theorized Model

**FIGURE 2:**
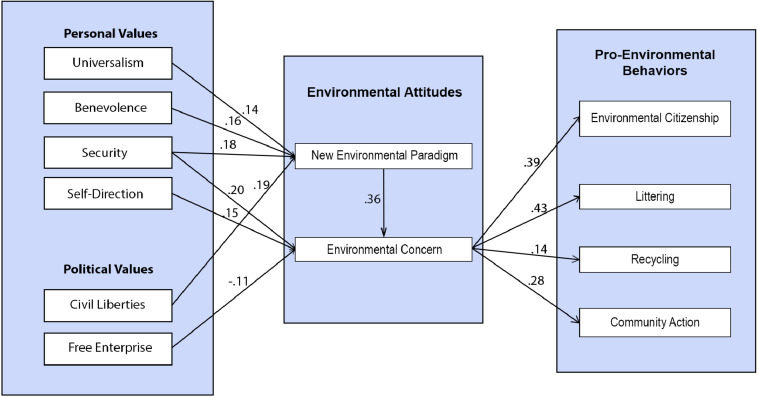
Path model: relationships between values, environmental attitudes, and pro-environmental behaviors.

The model with standardized regression values are presented in Figure 2. Our hypothesized model assessing the relationships between personal values, political values, environmental attitudes (NEP, EC), and pro-environmental behaviors explained 24% of variance in NEP, 32% of variance in EC, 15% of variance in Environmental Citizenship, 19% of variance in Littering, 2% of variance in Recycling, and 8% of variance in Community Action. The analysis revealed a good fit for the model χ^2^/df = 2.15; CFI = 0.95; RMSEA = 0.061; SRMR = 0.06, *p* < 0.001. NEP was significantly predicted by Civil Liberties (β = 0.20, *p* < 0.001), Universalism (β = 0.14, *p* = 0.03), Benevolence (β = 0.16, *p* = 0.02), and Security (β = 0.19, *p* = 0.003). Environmental Concern was significantly predicted by Security (β = 0.19, *p* < 0.001), Free Enterprise (β = −0.11, *p* = 0.03), Self-Direction (β = 0.15, *p* = 0.005), and NEP (β = 0.36, *p* < 0.001). These findings partially confirm hypotheses 2 and 3. Environmental Concern mediated the relationship between values and pro-environmental behavior, being a significant predictor of Environmental Citizenship (β = 0.39, *p* < 0.001), Littering (β = 0.43, *p* < 0.001), Recycling (β = 0.14, *p* = 0.01), and Environmental Community Action (β = 0.28, *p* < 0.001); NEP was not predicting any pro-environmental behavior. These findings partially confirm hypothesis 1, regarding environmental concern, but reject it regarding NEP.

### Creating Value-Based Profiles

To analyze the emergence of separate case profiles in the dataset, a K-Means cluster analysis was performed using the independent variables. It was decided to leave a three-cluster solution based on the number of iterations needed to bring the change in cluster centers to 0 in each group. The ANOVA test showed that each variable except for the value of Power (F = 0.77, *ns*) made a significant contribution to the formation of clusters.

Table 3 presents the group means and F-ratio of differences between the clusters. The first, youngest [age groups: 75 (18–24), 13 (25–44), 4 (45 and older); settlement sizes: 59 (cities of republican significance), 17 (regional centers), 13 (small and medium towns), 3 (villages); language: 19 (Kazakh), 74 (Russian); gender: 44 (female), 47 (male)], predominantly Russian-speaking cluster from larger cities with nearly equal number of female and male participants was called “Progressive.”

**TABLE 3:** Mean scores of clusters and F-ratio of differences between clusters.

	Mean, progressive, *N* = 93	Mean, apathetic, *N* = 63	Mean, traditionalist, *N* = 149	F
Power (0–8)	4.37	4.05	4.01	0.77
Achievement (0–8)	6.57	4.00	6.05	37.57***
Hedonism (0–8)	5.29	3.95	4.79	6.49**
Stimulation (0–8)	6.02	3.81	6.41	43.68***
Self-direction (0–8)	7.37	4.44	7.33	136.1***
Universalism (0–8)	7.03	4.38	7.35	114.51***
Benevolence (0–8)	6.78	4.46	7.70	142.86***
Tradition (0–8)	3.55	4.22	7.13	138.92***
Conformity (0–8)	4.29	4.46	7.21	94.4***
Security (0–8)	6.18	4.75	7.63	88.86***
Foreign military intervention	2.73	2.99	3.07	2.91*
Free enterprise	2.89	2.58	2.45	4.97**
Traditional morality	2.03	3.58	3.95	182.77***
Equality	3.79	3.84	4.35	14.78***
Immigration	3.39	2.88	2.8	14.56***
Blind patriotism	1.99	3.33	3.9	120.68***
Civil liberties	4.46	3.89	4.64	21.62***
Law and order	2.24	3.2	3.21	42.86***
NEP^a^	3.95	3.49	4.06	21.64***
EC (1–7)^b^	5.77	5.05	6.14	18.73***
Environmental citizenship	3.84	3.25	3.57	5.87**
Community action	2.19	2.63	2.99	10.08***
Recycling	2.6	2.93	2.62	1.32
Littering	4.73	4.22	4.66	11.45***

All scales 1–5, if otherwise is not specified. ^*a*^NEP, New Environmental Paradigm. ^*b*^EC, Environmental Concern. *p < 0.05; **p < 0.01; ***p < 0.001.

The second cluster [age groups: 29 (18–24), 24 (25–44), 9 (45 and older); settlement sizes: 15 (cities of republican significance), 16 (regional centers), 17 (small and medium towns), 15 (villages); language: 32 (Kazakh), 31 (Russian); Gender: 42 (female), 21 (male)], was called “Apathetic.”

**FIGURE 3:**
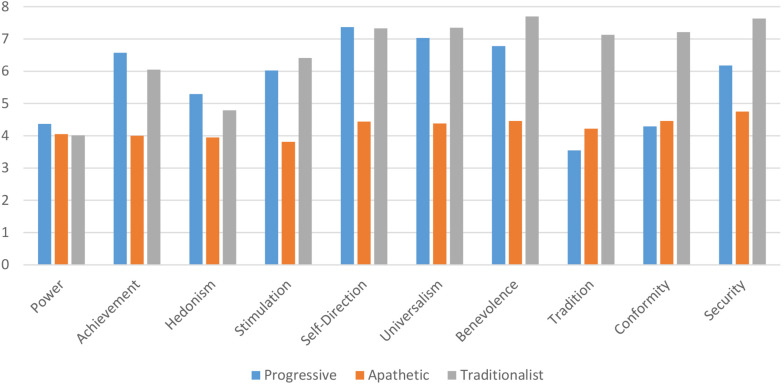
Mean scores of clusters on personal values.

**FIGURE 4:**
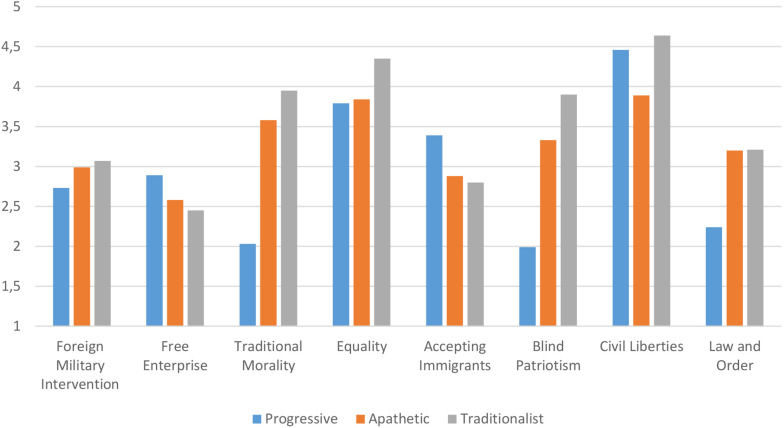
Mean scores of clusters on political values.

The third [age groups: 59 (18–24), 62 (25–44), 28 (45 and older); settlement sizes: 48 (cities of republican significance), 44 (regional centers), 36 (small and medium towns), 21 (villages); language: 63 (Kazakh), 86 (Russian); gender: 97 (female), 52 (male)], oldest cluster from both larger and smaller cities was named “Traditionalist.” Further differences between clusters can be found in Figures 3, 4.

To help better understand the differences between the clusters, Bonferroni’s *post-hoc* test was carried out. As was pointed out before, the three clusters had a non-significant difference on the value of Power. Regarding personal values of Achievement, Hedonism, Stimulation, Self-Direction, and Universalism, the Apathetic cluster had a significantly lower score (*p* < 0.001) than the other two clusters which had non-significantly different scores from each other. On personal values of Tradition and Conformity, the Traditionalist cluster had significantly higher scores (*p* < 0.001) than Progressive and Apathetic clusters, which also had non-significant differences between each other. On the values of Benevolence and Security, all three clusters showed significant differences in scores (*p* < 0.001). Traditional cluster had the highest scores on Benevolence and Security, followed by Progressive cluster, while the Apathetic cluster had the lowest scores on these values.

Bonferroni’s test revealed differences between Progressive and Traditionalist clusters on the value of Foreign Military Intervention (*p* = 0.052), with Traditionalists favoring military intervention more than Progressives; the Apathetic cluster, having scored in the middle, was non-significantly different from both clusters. An opposite tendency was found regarding the value of Free Enterprise, Progressives scoring significantly higher than Traditionalists (*p* = 0.006), but non-significantly higher than the Apathetic cluster. Traditionalists had significantly higher scores on Traditional Morality and Blind Patriotism, the Apathetic cluster also scored significantly higher on these values, than Progressives. Traditionalists also scored significantly higher than the other groups on Equality, while Progressive and Apathetic clusters did not differ significantly. Progressives had significantly higher scores on Accepting Immigrants than the other two groups; the scores of Traditionalists and the Apathetic group did not differ significantly. The Apathetic group had the lowest scores on Civil Liberties, demonstrating significant difference from the other groups’ scores. Progressive cluster had significantly different scores on Law and Order, having scored lower than both Traditionalist and Apathetic clusters, two of which did not differ on this value.

**FIGURE 5:**
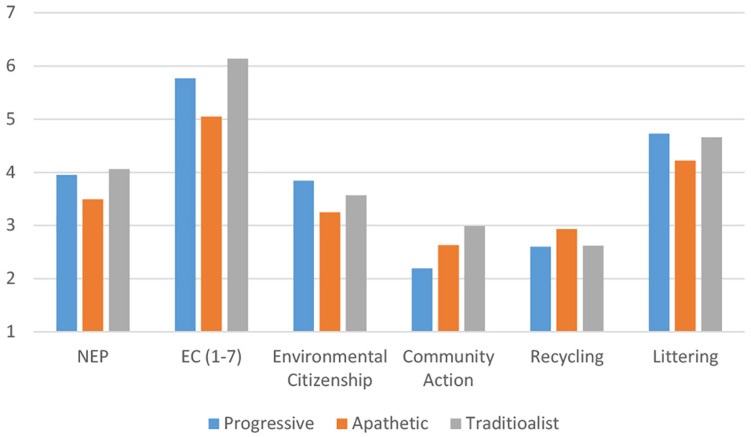
Mean scores of clusters on New Environmental Paradigm (NEP), Environmental Concern (EC), and pro-environmental behaviors.

The ANOVA revealed significant differences between clusters on all dependent variables. Bonferroni’s *post-hoc* test showed that the Apathetic cluster had significantly lower scores on EC, NEP, and Littering (*p* < 0.001) than Progressive and Traditionalist clusters. The latter two clusters did not show significantly different scores on EC and NEP. Progressive and Apathetic clusters had significantly different scores on Environmental Citizenship (*p* = 0.002), with the latter engaging in this behavior less often. Progressive cluster had a significantly lower score than the Traditionalist cluster on Community Action (*p* < 0.001). The clusters did not have significant differences on Recycling. Figure 5 presents the differences between clusters on NEP, EC, and pro-environmental behaviors.

## Discussion

### Environmental Concern, NEP, and Pro-environmental Behaviors

The aim of this research was to find how basic personal and political values predicted environmental attitudes, and how environmental attitudes predicted pro-environmental behavior in Kazakhstan. Results were mixed in terms of replicating the previous findings.

We studied how Environmental Concern and The New Environmental Paradigm influenced the four types of pro-environmental behavior. In our study, while Environmental Concern was the strongest predictor of all four behavior types, NEP did not have a significant effect on any of them. This finding is in line with other studies that compared the effect of both Environmental Concern and NEP on pro-environmental behavior (Berenguer et al., 2005; Landry et al., 2018). Our model findings can be explained partly by paralleling it with Value-Belief-Norm theory of pro-environmental behavior, which also showed that NEP influences pro-environmental behavior through other variables (Stern et al., 1999). Our model also showed that tendency: it is not NEP alone, but the presence of concern, or understanding of the bad impact, that leads to behavior.

### The Role of Schwartz’s Basic Personal Values in Predicting Environmental Attitudes and Behavior

One of the unexpected findings was the absence of effect of personal values of Self-Transcendence, Universalism, and Benevolence on Environmental Concern. In addition, the values of Self-Enhancement showed a pattern different from that observed in previous research: the value of Power did not predict Environmental Concern negatively, demonstrating no significant effect. These findings are not in line with previous research (Stern and Dietz, 1994; Poortinga et al., 2004; Hurst et al., 2013).

Different from previous findings in the Western cultures, our results are in line with the research conducted in other post-Soviet countries. Two recent studies conducted in Russia did not find an effect of Altruistic values on pro-environmental variables (Sautkina, 2019; Ünal et al., 2019). Our results may suggest that for Kazakhstanis, similarly to people from other developing countries (Hurst et al., 2013), the environmental problems are not necessarily related to caring about others.

In addition to this, we found a significant positive effect of the value of Security on Environmental Attitudes. This result is particularly significant, given that those living in the developing countries are increasingly more likely to become victims of negative consequences of environmental and climatic change. Sociological polls carried out in Kazakhstan showed people’s concern for their health (Freedman, 2009), which can be mainly related to issues of personal safety, in other words—the value of Security.

These two findings, the absence of effect of Self-Transcendence, Universalism, and Benevolence on Environmental Concern and the role of the value of Security, may suggest that while in developed nations environmental issues are related to “others” that need to be cared for, in Kazakhstan, a developing country, environmental issues are related to one’s personal safety, therefore being important at an entirely different level. Comparing, once again, the context of Kazakhstan with that of Russia, Soyez (2012) concluded that unlike for people of other countries (e.g., the United States, Canada, Germany, and Australia), the issues of nature protection were a matter of survival for Russians.

Another finding, the influence of the value of Self-Direction, is in line with the previous research which found a positive effect of Openness to Change values on environmentalism (Schultz et al., 2005). This also supports the findings of the study conducted on Russian youth (Zibenberg et al., 2018), reflecting the social processes in the post-Soviet countries where environmentalism and pro-environmental movements are relatively new and gaining traction. The value of Self-Direction promotes openness to new ideas and new movements, thereby also promoting support and awareness regarding the environmental agenda. Younger generations of Kazakhstani, due to the growing internationalization, tend to be more individualist and more aware about global environmental issues. In addition, the low level of environmental policy development in many parts of the post-Soviet world may contribute to explaining this relationship: environmentalists may rely less on the governments to solve the environmental problems (Sautkina, 2019).

We also found that several values had a direct influence on pro-environmental behaviors. Benevolence predicted Littering behavior, which may be an indicator of care for nature. Benevolence is close in its meaning to Altruistic values, defined by caring about other people. Though Altruistic values are related to biospheric values and environmental concern, they have not been previously found to predict pro-environmental behavior directly (De Groot and Steg, 2007; Lee et al., 2014). This may be due to the fact that more hard-to-perform types of pro-environmental behavior, such as recycling or sustainable consumption, were measured in previous research (Lee et al., 2014). We conclude that littering avoidance, a much easier behavior, could be influenced by values directly.

In our study, Universalism was found to increase Environmental Citizenship, while Conformity decreased this behavior. Environmental Citizenship is a social act of learning about the issue and talking about the environment, thereby influencing others. People with Universalist values feel the importance of this issue, therefore they engage in Environmental Citizenship behavior. Conformity, on the other hand, is against the expression of personal values and feelings and therefore, suppresses the active social behavior. In a cross-cultural study by Tam and Chan (2017), it was found that in countries with higher levels of individualism and looseness, environmental concern led to pro-environmental behavior. Therefore, such cultural variates as collectivism, which often implies conformity (Bond and Smith, 1996), may reduce pro-environmental behavior.

Of a greater interest, though, is the positive effect of Power on Community Action. On the other hand, Community Action was reported more often by rural, Kazakh-speaking, older respondents. Organizing these community events and participating in them was a practice that originates from the Soviet system, where such events tended to be obligatory. There is a need for further research in this area with more detailed and culture-specific measures of Community Action.

### The Role of Core Political Values in Predicting Environmental Attitudes and Behavior

In this study, we investigated how political values predicted environmental concern and pro-environmental behaviors in a polarization-free political context. Our findings demonstrate that there are certain aspects of liberal and conservative ideology, such as the political values of Civil Liberties and Free Enterprise, that can predict the Environmental Concern.

The core political value of Civil Liberties positively predicted environmental attitudes, being a stronger predictor of NEP, than of Environmental Concern. Free Enterprise, on the other hand, decreased environmental concern. This finding does not correspond to previous cross-national findings (Nawrotzki, 2012; Kim and Shin, 2015; Ziegler, 2017). While in these studies, liberal and conservative ideologies were measured directly, in our study, we measured values related to political ideologies. Political values reflect multiple facets of political ideologies and our finding that there is a certain aspect within a political ideology which predicts environmental attitudes suggests the need to study this relationship in more detail. For example, Thorisdottir et al. (2007) found that conservatism has two dimensions in Eastern European countries—economic (this includes freedom of personal enterprise) and social (this includes traditionalism and conservation). Our findings show that it is not social, but economic conservatism that is negatively related to environmentalism. In addition, Free Enterprise value implies an unfettered pursuit of own success and wealth (Schwartz et al., 2010), which in relation to the environmental topic, is likely to signify dominance over nature, uncontrollable exploitation of its resources and overconsumption, ultimately representing principles that are in contradiction with concern for the environment.

Higher scores on Civil Liberties also predicted higher levels of NEP and less of Littering, which corresponds to the findings regarding the main influence of ideologies on pro-environmental behaviors (Cruz, 2017). Quite surprisingly, Law and Order was a negative predictor of Littering behavior. This might be due to the nature of questions, which, instead of asking about personal responsibility, emphasize the main role of the government and enforcement agencies in maintaining social order.

Another interesting finding was a positive influence of the value of Accepting Immigrants on Recycling. As proposed by Schwartz et al. (2010), Accepting Immigrants reflects the personal value dimension of Openness to Change, which contains Self-Direction. Recycling behavior in general is a new way of living in Kazakhstan, emerging only lately in urban areas where adequate facilities have been made available. Therefore, this relationship can be explained by a third variable (i.e., urban living, implying the availability of recycling facilities), as people who are more accepting of immigrants tend to be progressive and live in bigger cities.

In sum, the use of political values scale in the present study has shown its potential to significantly contribute to the development of the evidence base on predictors of environmentalism. In future research on its political dimension, we suggest measuring political values not only in polarization-free contexts but also in countries where polarization is traditionally being discussed, to capture a more realist spectrum of peoples’ views. In conclusion, what our findings suggest is that measuring the dichotomy: liberal vs. conservative ideology can be simplistic and does not offer the respondents the opportunity to express their actual, more diverse, and ultimately more realist, opinions.

### Value-Based Environmental Profiles

The cluster analysis revealed three distinct groups of people that we called “Progressive,” “Apathetic,” and “Traditionalist.” Despite the differences in their values and socio-demographic characteristics, Progressive and Traditionalist clusters were the most pro-environmental, meaning that engaging in pro-environmental behavior can be determined by different value types.

The first, youngest group that we labeled “Progressive” is the most liberal group. Despite being liberal, this profile scored highly on Free Enterprise. Researchers who have been studying Kazakhstan called the youth of the post-Soviet Era the “Nazarbayev Generation” (Junisbai and Junisbai, 2020) stating that the generation born during the years of the presidency of Nursultan Nazarbayev (1991–2019) was different from the generation of their parents. The Nazarbayev Generation accepts economic inequalities, relies less on the support of the state and is less influenced by traditional family values (Junisbai and Junisbai, 2020). The “Progressives” are concerned about the environment and talk about the environment more than any other group. Interestingly, they almost do not participate in environmental community action which is more characteristic of state-supporting or tradition-valuing participants. This may be due to the highly government-imposed nature of community action in Kazakhstan, which does not attract this progressive youth.

The second group which we called “Apathetic” is characterized by their avoidance of expressing certain opinions. Political apathy has already been described in Kazakhstan (Bowyer, 2008). Mainly representing the rural population, this group still has high levels of environmental concern, but scores lower on action, i.e., pro-environmental behavior.

The third group named “Traditionalist” had high scores on almost each “socially desirable” variable. Their scores may represent what is considered good or bad in the Kazakh society. Their answers, probably, are not as much their own beliefs, but the beliefs that are most popular, or acceptable, within the culture. “Traditionalists” are not “classic” western conservatives, as they do not value Free Enterprise. Rather, they prefer to rely on the government and demand economic equality, which is in line with other findings in Eastern European samples (Thorisdottir et al., 2007). Another notable trait is that they do not accept immigrants, at the same time valuing equality and liberties. These discrepancies show the nature of their endorsement of liberalism. Liberal values of Equality and Civil Liberties for them may be nationalistic, it is not about protecting minorities or “others,” it is about liberating the Kazakh nation.

Consistently, “Traditionalists” score the highest on environmental concern and engage, to a great extent, in environmental citizenship and community action behaviors. Caring about the environment may also be a desirable trait in Kazakh culture, as this link was found in other collectivist cultures (Karp, 1996; Rice, 2006). As their traditionalism does not contradict, but endorses environmentalism, we can assume that emphasizing traditional values may be an important strategy for promoting environmentalism in Kazakhstan.

In this research, the cluster groups of values and their scores on environmentalism were investigated for the first time. Though pro-environmental behavior clusters have been analyzed before (Elgaaied, 2012; Lind et al., 2015; Holmes et al., 2019), our findings expand this body of research suggesting that groups forming clusters based on behavior may also differ in their value structure that influences environmentalism.

Despite the differences in their values and beliefs, all three groups had considerably high levels of Environmental Concern. They differed substantially in their pro-environmental behaviors; however, all three groups had equally low levels of Recycling behavior. These results give promising implications for policy-makers regarding raising the awareness and promotion of pro-environmental culture. Environmental campaigners could appeal to progressive youth and traditionalist older generations by using different rhetoric and be equally effective.

## Limitations and Future Research Directions

One of the main limitations of this research is the lack of equivalence between the scales in two languages—Kazakh and Russian. This discrepancy may have influenced the results on the relationships between the studied constructs. The Kazakh language has an entirely different nature from English; therefore, it was challenging to reach the perfect consistency between the two versions of the questionnaire. Nevertheless, the linguistic consistency of the research tools was approved by a native English speaker and was as close to the original as possible.

The studied sample was not fully representative of the Kazakh population in terms of age, regions, income, education level, etc. which may have biased the results.

Different scales were used to measure different types of pro-environmental behavior, i.e., Littering and Recycling vs. Environmental Citizenship and Community Action. The absence of appropriate methodological tools in Russian and Kazakh calls for adaptation of the existing pro-environmental behavior scales and the creation of those more adapted to the national contexts (see Ivanova et al., 2020).

This study relied on self-report measures which have frequently been found to bias study findings. This calls for future studies using objective measures, as well as methodological triangulations in order to converge, corroborate, and complement data obtained from various methods (Johnson et al., 2007; Thomas et al., 2019).

Future research should consider measuring political values not only in polarization-free contexts but also where political polarization exists, to capture a fuller spectrum of political views.

It would be beneficial to further investigate the relationship between, on the one hand, values and political values, and, on the other, environmentalism in cross-cultural contexts.

The role of Self-Transcendence vs. Conservation (in particular, Security value) in determining environmentalism in developing countries that are particularly vulnerable to the consequences of environmental change need to be further explored.

Finally, further research using clusters and profiles related to environmentalism seems theoretically and practically promising.

## Conclusion

Our research has contributed to enhancing the evidence on the influence of basic personal and core political values on environmental attitudes and pro-environmental behavior. We found that environmental attitudes—NEP and environmental concern—were predicted by different sets of values, which emphasizes the need to differentiate between them. The value of Security predicted both environmental attitudes, overshadowing the values of Self-Transcendence and Self-Enhancement, traditionally considered the main predictors of environmentalism. While the value of Self-Direction predicted environmental concern, Universalism and Benevolence predicted NEP. This emphasizes that the value structure related to environmentalism is different in Kazakhstan, showing that perception of environmental threats is unrelated to self-transcendence and caring for others, but rather, represents an issue of independent thinking and action, and personal security in the face of environmental threats. This underlines the importance of further cross-cultural research regarding the role of values in determining environmentalism.

The study of political values added a new, significant and more nuanced, prediction of environmental attitudes, while also replicating tendencies found in the Western democracies. Specifically, the political value of Civil Liberties predicted NEP positively, and Free Enterprise predicted environmental concern negatively. These findings expand the evidence base on the determinants of environmental attitudes and pro-environmental behaviors, demonstrating a crucial cultural influence on the relationship between these variables. This issue is also of practical importance: reflecting two aspects of a nation (socio-cultural and political), they can help policy-makers develop pro-environmental campaigns that align with the national value systems.

Finally, three distinct profiles of respondents with different values were identified, differing in their environmental attitudes, pro-environmental behavior and socio-demographic characteristics.

## Data Availability Statement

The raw data supporting the conclusions of this article will be made available by the authors, without undue reservation.

## Ethics Statement

The studies involving human participants were reviewed and approved by the Institutional Review Board of the National Research University Higher School of Economics, Russia. Written informed consent for participation was not required for this study in accordance with the national legislation and the institutional requirements.

## Author Contributions

FA designed the study, collected, analyzed, and interpreted the data, and led on the writing of this article. ES supervised the work and made significant contributions to the study design, data analysis, and interpretations, as well as to the writing of this article. Both authors contributed to the article and approved the submitted version.

## Conflict of Interest

The authors declare that the research was conducted in the absence of any commercial or financial relationships that could be construed as a potential conflict of interest.
