# Supplementary material for: The Role of Personal and Political Values in Predicting Environmental Attitudes and Pro-environmental Behavior in Kazakhstan
Source: Front Psychol. 2020 Dec 23;11:584292. doi: 10.3389/fpsyg.2020.584292 (PMC7793664; doi:10.3389/fpsyg.2020.584292)
Supplement: Supplementary file 3 [file Table_3.DOCX]

Supplement File 3

Questionnaires used in the study

| Original English version | Russian version | Kazakh version |
| --- | --- | --- |
| **Personal Values** (Lindeman & Verkasalo, 2005)  Please, rate the importance of the following values as a life-guiding principle for you. Use the 8-point scale in which 0 indicates that the value is opposed to your principles, 1 indicates that the values is not important for you, 4 indicates that the value is important, and 8 indicates that the value is of supreme importance for you.  POWER (social power, authority, wealth)  ACHIEVEMENT (success, capability, ambition, influence on people and events)  HEDONISM (gratification of desires, enjoyment in life, self-indulgence)  STIMULATION (daring, a varied and challenging life, an exciting life)  SELF-DIRECTION (creativity, freedom, curiosity, independence, choosing one's own goals)  UNIVERSALISM (broad-mindedness, social justice, a world at peace, equality, wisdom)  BENEVOLENCE (helpfulness, honesty, forgiveness, loyalty, responsibility)  TRADITION (respect for tradition, humbleness, accepting one's portion in life, devotion, modesty)  CONFORMITY (obedience, honoring parents and elders, self-discipline, politeness)  SECURITY (national security, family security, social order, cleanliness, reciprocation of favors) | Пожалуйста, оцените важность следующих ценностей в вашей жизни. Используйте 8-балльную шкалу, в которой 0 означает, что ценность противоречит вашим принципам, 1 означает, что ценность не важна для вас, 4 означает, что ценность важна, а 8 означает, что ценность имеет для вас наивысшее значение.  ВЛАСТЬ (социальная власть, авторитет, богатство)  ДОСТИЖЕНИЕ (успех, способности, амбиции, влияние на людей и события)  ГЕДОНИЗМ (удовлетворение желаний, наслаждение жизнью, самоугождение)  СТИМУЛЯЦИЯ (смелость, разнообразная и полная вызовов жизнь, захватывающая жизнь)  САМОСТОЯТЕЛЬНОСТЬ (креативность, свобода, любознательность, независимость, выбор своих целей)  УНИВЕРСАЛИЗМ (широкий взгляд на мир, социальная справедливость, мир во всем мире, равенство, мудрость)  БЛАГОЖЕЛАТЕЛЬНОСТЬ (готовность помочь, честность, прощение, верность, ответственность)  ТРАДИЦИЯ (уважение к традициям, смирение, принятие своей доли в жизни, преданность, скромность)  КОНФОРМИЗМ (послушание, почитание родителей и старших, самодисциплина, вежливость)  БЕЗОПАСНОСТЬ (национальная безопасность, семейная безопасность, социальный порядок, чистота, взаимность оказания услуг) | Келесі құндылықтардың өмірлік ұстаным ретінде өзіңіз үшін маңыздылығын бағалаңыз. Сегіз балдық шкалада 0 мәні құндылықтың ұстанымдарыңызға қайшы келетіндігін, 1 Сіз үшін маңызды емес екенін, 4 маңызды екенін және 8 ең маңыздысы екенін білдіреді.  КҮШ (әлеуметтік ықпал, билік, байлық)  ЖЕТІСТІК (жетістік, қабілет, өршілдік, адамдар мен оқиғаларға әсер ете алу)  ГЕДОНИЗМ (тілектерді қанағаттандыру, өмірден ләззат алу, өзінің ықыластарын қанағаттандыру)  ЫНТАЛАНДЫРУ (батылдық, жан-жақты және әртүрлі сындарға толы өмір, қызықты өмір)  ЖЕКЕ БАҒЫТ (шығармашылық, еркіндік, қызығушылық, тәуелсіздік, өз мақсаттарын таңдау)  УНИВЕРСАЛИЗМ (кең өрістілік, әлеуметтік әділеттілік, бейбітшілік, теңдік, даналық)  АҚ НИЕТТІЛІК (мейірімділік, шындық айту, кешірімділік, адалдық, жауапкершілік)  ДӘСТҮРЛІЛІК (дәстүрді құрметтеу, кішіпейілділік, тағдырға көну, адалдық, қарапайымдылық)  БОЙСҰНУ (кішіпейілділік, ата-ана мен үлкенді сыйлау, ұстамдылық, сыпайылық)  ҚАУІПСІЗДІК (ұлттық қауіпсіздік, отбасы қауіпсіздігі, әлеуметтік тәртіп, тазалық, игі ниеттілік) |
| **Core Political Values** (Schwartz et al., 2010)  Please rate your agreement with each statement. 1 – totally agree, 5 – completely disagree:  Political measures to increase security should be promoted at this time, even if it could mean sacriﬁcing the freedom of citizens  The police should have more powers so they can protect us better against crime  There should be limits on the freedom of speech of people who threaten society  The most important thing for our country is to maintain law and order  It’s right for the government to take restrictive measures on civil liberties to guarantee the security of citizens  Order has to be preserved at any cost, even if this could reduce civil liberties  It would be a good idea to limit the liberty of expression if this can guarantee more order  This country would have many fewer problems if there were more emphasis on traditional family ties  It is extremely important to defend our traditional religious and moral values  Homosexual couples should have the same rights as married couples  The right to life has to be guaranteed by law from the moment of conception (deleted)  Newer lifestyles are contributing to the breakdown of our society  If people were treated more equally in this country, we would have many fewer problems  Our society should do whatever is necessary to make sure that everyone has an equal opportunity to succeed  The government should do more to guarantee an equal distribution of resources between rich and poor  The government should take responsibility to provide free health care to all citizens  Going to war is sometimes the only solution to international problems (deleted)  War is never justiﬁed (deleted)  Italy should contribute forces to international peace-keeping efforts  Italy should join other democratic nations in sending troops to ﬁght dangerous regimes  Any act is justiﬁed to ﬁght terrorism (deleted)  It would be a good idea to privatize all of the public enterprises  The less government gets involved with business and the economy, the better off this country will be  There should be more incentives for individual initiative even if this reduces equality in the distribution of wealth (deleted)  All high school and university education should be made private rather than controlled and supported by the government  It is extremely important to respect the freedom of individuals to be and believe whatever they want  The most important thing for our country is to defend civil liberties  The right to individual freedom is inviolable and has to be maintained at all cost  It is unpatriotic to criticize this country  It’s a duty of all citizens to honor the country  I would support my country right or wrong  People who come to live here from other countries generally make Italy a better place to live  People who come to live here from other countries generally take jobs away from Italian workers  People who come to live here from other countries make Italy’s cultural life richer | Russian version from (Kholod, 2016)  Пожалуйста, оцените степень согласия с каждым из утверждений, 1 - абсолютно не согласен, 5 - абсолютно согласен.  Политические меры по усилению безопасности должны поощряться, даже если это может угрожать свободе граждан  Чтобы полиция могла лучше защищать нас от преступлений, она должна иметь больше полномочий  Должны существовать ограничения на свободу слова у людей, угрожающих обществу  Поддержание законов и правопорядка - самое важное для нашей страны  Применение ограничительных мер гражданских свобод для гарантии безопасности граждан является правильным решением для правительства  Порядок должен быть сохранен любой ценой, даже если он может ограничить гражданскую свободу  Ограничение свободы слова может быть лучшим решением, если оно будет гарантировать больше порядка  В нашей стране было бы меньше проблем, если бы мы поддерживали традиционные семейные узы  Крайне важно защищать наши традиционные религиозные и моральные ценности  Гомосексуальные пары должны иметь такие же права, как и гетеросексуальные  Право на жизнь должно быть гарантировано законом с момента зачатия  Новые стили жизни способствуют упадку нашего общества  Мы бы имели намного меньше проблем, если бы в этой стране обходились со всеми одинаково  Нашему обществу следует делать все необходимое, дабы убедиться, что каждый имеет равные возможности для достижения успеха  Правительство должно делать еще больше, чтобы гарантировать равное распределение средств между богатыми и бедными  Правительство должно взять на себя ответственность за оказание бесплатной медицинской помощи всем гражданам  Развязывание войны иногда является единственным решением международных проблем  Война никогда не может быть оправдана  Казахстан должен вкладывать силы в международные миротворческие усилия  Казахстан должен присоединиться к другим демократическим государствам в отправке войск на борьбу с опасными режимами  Любые действия в борьбе с терроризмом оправданы  Было бы хорошей идеей приватизировать все государственные предприятия  Чем меньше правительство привлекается к бизнесу и экономике, тем лучше для страны  Должно существовать больше поощрений личной инициативы даже, если это уменьшит равноправие в распределении материальных ценностей  Всё школьное и университетское образование должно быть частным, а не контролироваться и поддерживаться правительством  Крайне важно уважать свободу людей верить в то, во что они хотят верить  Самое важное для нашей страны — это защищать гражданские свободы  Право на свободу личности неприкосновенно и должно быть сохранено любой ценой  Критиковать эту страну – непатриотично  Долг каждого гражданина - почитать свою страну  Я бы поддерживал свою страну при любых обстоятельствах – права она или нет  Люди, которые приезжают жить в Казахстан из других стран, в большинстве случаев делают его лучшим местом для жизни  Люди, которые приезжают жить в Казахстан из других стран, в большинстве случаев отбирают работу у казахстанских работников  Люди, которые приезжают жить в Казахстан из других стран, делают его культурную жизнь богаче | Төменде жазылған пікірлермен қаншалықты келісетініңізді белгілеңіз. 1 - толығымен келісемін, 5 - толығымен келіспеймін  Азаматтардың бостандығы құрбан етілсе де, қауіпсіздікті арттыру мақсатында саяси іс-шараларды насихаттау қажет.  Полиция бізді жеткілікті түрде қорғау үшін көбірек өкілеттікке ие болғаны жөн  Қоғамға қауіп төндіретін адамдардың сөз бостандығына шектеулер қою керек  Біздің еліміз үшін ең бастысы - заңдылық пен тәртіпті сақтау  Азаматтардың қауіпсіздігін қамтамасыз ету үшін үкімет азаматтық бостандықты шектейтін шаралар қабылдаса болады  Тәртіп кез-келген жағдайда, тіпті азаматтық бостандықты шектесе де сақталуы керек  Тәртіпті қамтамасыз ету мақсатында сөз бостандығын шектеген дұрыс  Дәстүрлі отбасылық қатынастарға көбірек көңіл бөлінгенде бұл елдің мәселелері де әлдеқайда азырақ болар еді  Дәстүрлі діни және моральдық құндылықтарымызды қорғау өте маңызды  Гомосексуалдық жұптар дәстүрлі ерлі-зайыптылармен бірдей құқықтарға ие болулары керек  Өмір сүру құқығы бала біту сәтінен заңмен қамтамасыз етілген жөн  Жаңа өмір салттары біздің қоғамның ыдырауына ықпал етеді  Адамдардың арасында теңдік орнағанда, мемлекет ішінде мәселелер де әлдеқайда азырақ болар еді.  Әр адам жетістікке жету жолында бірдей мүмкіндікке ие болу үшін біздің қоғам қолдан келгенше барлығын істеуі керек  Үкімет байлар мен кедейлер арасында ресурстардың тең бөлінуіне кепілдік беруі керек  Үкімет барлық азаматтарға ақысыз медициналық көмек көрсету бойынша жауапкершілікті өз мойнына алуы керек  Соғысқа бару кейде халықаралық мәселелерді шешудің жалғыз әдісі болып табылады  Соғысты ақтау мүмкін емес  Қазақстан халықаралық бітімгершілік күштерге өз үлесін қосуы керек  Қазақстан қауіпті режим орнаған жерлерге әскер жіберетін демократиялық елдердің қатарына қосылуы тиіс  Терроризммен күрес жолында барлық шараларды қолданса болады  Барлық мемлекеттік кәсіпорындарды жекешелендірген дұрыс болар еді  Үкімет бизнес пен экономикаға неғұрлым аз араласса, бұл елдің жағдайы соғұрлым жақсы болады  Жеке бастаманы ынталандыруға көбірек көңіл бөлінуі керек. Бұл тіпті байлықтың тең емес бөлінген жағдайында болуы мүмкін.  Барлық орта және жоғары оқу орындары үкіметтің бақылауында емес, жеке тұлға бақылауына өтуі керек  Жеке адамдардың бостандықтарын құрметтеу және сенімдерін құрметтеу өте маңызды  Біздің еліміз үшін ең бастысы - азаматтық бостандықты қорғау  Жеке бас бостандығына қол сұғылмауы тиіс және ол қай жағдайда болсын сақталған жөн.  Бұл елді сынға алу патриоттық тұрғыдан бұрыс әрекет.  Барлық азаматтар елді құрметтеу керек  Мен өз елімді кез келген жағдайда қолдаймын әрі жақтаймын  Басқа елдерден көшіп келген адамдар Қазақстанның жағдайын жақсартуда  Басқа елдерден қоныс аударған адамдар қазақстандықтардың жұмыс орындарын «тартып алуда»  Басқа елдерден көшіп келген адамдар Қазақстанның мәдени өмірін байытуда |
| **New Environmental Paradigm** (Dunlap, 2000)  We are approaching the limit of the number of people the Earth can support  Humans have the right to modify the natural environment to suit their needs  When humans interfere with nature it often produces disastrous consequences  Human ingenuity will insure that we do not make the Earth unlivable (deleted)  Humans are seriously abusing the environment  The Earth has plenty of natural resources if we just learn how to develop them (deleted)  Plants and animals have as much right as humans to exist  The balance of nature is strong enough to cope with the impacts of modern industrial nations (deleted)  Despite our special abilities, humans are still subject to the laws of nature.  The so-called “ecological crisis” facing humankind has been greatly exaggerated (deleted)  The Earth is like a spaceship with very limited room and resources  Humans were meant to rule over the rest of nature  The balance of nature is very delicate and easily upset  Humans will eventually learn enough about how nature works to be able to control it (deleted)  If things continue on their present course, we will soon experience a major ecological catastrophe | Russian version from (Kryazh, 2013).  Мы приближаемся к предельному количеству людей, которое земля может поддержать  Люди имеют право изменять природную среду, чтобы удовлетворять свои потребности  Когда люди вмешиваются в природу, это часто приводит к бедственным последствиям  Человеческая изобретательность – гарантия того, что мы НЕ сделаем землю непригодной для жизни  Люди сильно злоупотребляют возможностью использовать окружающую среду  На земле вполне достаточно природных ресурсов, надо только научиться разрабатывать их  Растения и животные имеют такое же право на существование, как и люди  Природное равновесие достаточно устойчиво, чтобы справиться с воздействием современных промышленных стран  Несмотря на свои особые способности, люди все еще подчинены законам природы  Так называемый «экологический кризис», угрожающий человечеству, весьма преувеличен  Земля похожа на космический корабль с очень ограниченным помещением для ресурсов  Предназначение людей – управлять остальной природой  Природное равновесие очень хрупкое и легко может быть нарушено  Люди, в конечном счете, достаточно узнают о законах природы, чтобы быть в состоянии управлять ею  Если ситуация будет и дальше развиваться, как это происходит сейчас, мы скоро ощутим масштабную экологическую катастрофу | Біз жер қамти алатын адам санының шегіне жақындап келеміз  Адамзат өз қажеттіліктерін қанағаттандыру үшін табиғи қоршаған ортаны өзгертуге құқығы бар  Адамзаттың табиғатқа қарсы әрекет етуі, көбінесе апатты жағдай салдарын туғызып жатады  Адамзаттың тапқырлығы біз жерді өмір сүруге жарамсыз ортаға айналдырмайтынымызға кепіл  Адамдзат қоршаған ортаға зиян тигізуде  Егер біз оларды дамытуды үйренсек, Жерде көптеген табиғи ресурстар бар  Адамзат қаншалықты өмір сүруге құқылы болса, өсімдіктер мен жануарлардың да соншалықты құқығы бар  Табиғат тепе-теңдігі заманауи индустриялық елдердің әсерін жеңу үшін жеткілікті мықты.  Ерекше қабілеттерімізге қарамастан, адамдар әлі күнге дейін табиғат заңдарына бағынады  Адамзаттың алдында тұрған «экологиялық дағдарыс» мәселесі аса сілтенуде  Жер өте шектеулі бөлмелері мен ресурстары бар ғарыш кемесі сияқты.  Адамзат табиғатты басқаруға арналған.  Табиғат тепе-теңдігі өте нәзік және оңай бұзылады.  Адамдар табиғатты басқара білу үшін оның қалай жұмыс істейтінін болашақта жеткілікті дәрежеде үйренеді.  Егер қазіргідей жалғаса беретін болса, жақын арада біз ауқымды экологиялық апаттың куәгері боламыз. |
| **Environmental Concern**  (Berenguer et al., 2005; Sautkina, 2019)  Please, evaluate how concerned you are with the state of environment, where 1 = not at all concerned, and 7 = extremely concerned.  To what extent are you concerned about the situation of the environment in your region?  To what extent are you concerned about the situation of the environment in general?  To what extent are you concerned about the situation of the environment in your country? | Russian version from (Sautkina, 2019)  Пожалуйста, оцените насколько вы обеспокоены экологической ситуацией, 1 – совершенно не беспокоит, 7 – крайне беспокоит.  В какой степени Вас беспокоит общая ситуация с экологией в вашем регионе?  В какой степени Вас беспокоит общая ситуация с экологией в мире?  В какой степени Вас беспокоит общая ситуация с экологией в Казахстане? | Сізді қоршаған ортаның жағдайы қаншалықты алаңдататынын белгілеңіз, 1 - мүлде алаңдамаймын, 7 - қатты алаңдаймын  Сіздің аймағыңыздағы қоршаған ортаның жағдайы Сізді қаншалықты алаңдатады?  Сізді Қазақстандағы қоршаған ортаның жағдайы қаншалықты алаңдатады?  Сізді (әлемдегі) қоршаған ортаның жағдайы қаншалықты алаңдатады? |
| **Pro-Environmental Behavior**  Please mark how often you do following activities. 1 - never, 5 - always.  Recycling (Kaiser, 1998)  Recycling dead batteries  Taking paper/newspapers/magazines, glass bottles, plastic bottles to recycling  Littering  Carrying litter with oneself until one finds a bin (Zero Waste Scotland, 2013)  In car or boat, throwing things out on the highways or waterways (reversed item (Schultz, 2009)  Environmental Citizenship (Alisat & Riemer, 2015)  In the last six months, how often, if at all, have you engaged in the following environmental activities and actions?” Items rated on a 5-point scale from 1 (never) to 5 (frequently).  Talked with others about environmental issues (e.g., spouse, partner, parent(s), children, or friends)  Used online tools (e.g., YouTube, Facebook, Wikipedia) to raise awareness about environmental issues  Environmental Community Action (Alisat & Riemer, 2015)  Participated in nature conservation efforts (e.g., planting trees, restoration of waterways) | Как часто вы делаете следующие вещи? Оцените ваши ответы по 5-балльной шкале от 1 (никогда) до 5 (всегда).  Утилизирую разряженные батареи  Сдаю в утилизацию бумагу / газеты / журналы, стеклянные бутылки, пластиковые бутылки  Ношу мусор с собой, пока не найду мусорную корзину  Когда еду в машине или лодке, выбрасываю мусор на шоссе или водные пути  За последние 6 месяцев, как часто вы участвовали в следующих экологических мероприятиях и действиях? Оцените ваши ответы по 5-балльной шкале от 1 (никогда) до 3 (иногда) до 5 (часто).  Обсуждал с другими людьми проблемы окружающей среды (например, супруг, партнер, родитель(и), дети или друзья).  Использовал онлайн платформы (например, YouTube, Facebook, Wikipedia) для повышения своей осведомленности об экологических проблемах.  Участвовал в природоохранных мероприятиях (например, посадка деревьев, восстановление водных путей). | Төмендегі іс-әрекеттерді қаншалықты жиі жасайтыныңызды белгілеңіз. 1 – ешқашан, 5 – әрқашан  Пайдаланылған батареяларды қайта өңдеуге жіберу  Қағаз, газет-журналдар, шыны бөтелкелер, банкалар, пластикалық бөтелкелерді қайта өңдеуге жарату  Қоқысты қоқыс жәшігін тапқанға дейін алып жүру  Көлікте немесе қайықта жолға немесе суға заттар лақтыру  Соңғы 6 ай ішінде төмендегі қоршаған ортаға қатысты іс-әректтерге (егер қатысқан болсаңыз) қаншалықты жиі қатыстыңыз? Жауабыңызды 5-баллдық шкаламен 1-ден 5-ке дейін бағалаңыз (мұнда 1 – ешқашан, 3 – кейде, 5 – жиі).  Қоршаған орта мәселелері туралы өзгелермен (мысалы, жұбайыммен, серіктесіммен, ата-анаммен, балаларыммен немесе достарыммен) сөйлестім.  Қоршаған орта мәселелері туралы хабардарлықты арттыру үшін онлайн құралдар қолдандым (мысалы, YouTube, Facebook, Wikipedia).  Табиғатты қорғау іс-шараларына қатыстым (мысалы, ағаш отырғызу, су жолдарын қалпына келтіру). |
